# Evaluation and comparison of one-step real-time PCR and one-step RT-LAMP methods for detection of SARS-CoV-2

**DOI:** 10.1186/s12879-024-09574-9

**Published:** 2024-07-09

**Authors:** Hooman Hanifehpour, Fatemeh Ashrafi, Elham Siasi, Shirzad Fallahi

**Affiliations:** 1 Department of Microbiology, Faculty of Biological Sciences, North Tehran Branch, Islamic Azad University, Tehran, Iran; 2 Hepatitis Research Center, Lorestan University of Medical Sciences, Khorramabad, Iran; 3 Department of Parasitology and Mycology, School of Medicine, Lorestan University of Medical Sciences, Khorramabad, Iran

**Keywords:** Rapid, Sensitive, Detection, SARS-CoV-2, One-step LAMP, One-step RT-qPCR

## Abstract

**Background:**

There is an increasing disease trend for SARS-COV-2, so need a quick and affordable diagnostic method. It should be highly accurate and save costs compared to other methods. The purpose of this research is to achieve these goals.

**Methods:**

This study analyzed 342 samples using TaqMan One-Step RT-qPCR and fast One-Step RT-LAMP (Reverse Transcriptase Loop-Mediated Isothermal Amplification). The One-Step LAMP assay was conducted to assess the sensitivity and specificity.

**Results:**

The research reported positive samples using two different methods. In the RT-LAMP method, saliva had 92 positive samples (26.9%) and 250 negative samples (73.09%) and nasopharynx had 94 positive samples (27.4%) and 248 negative samples (72.51%). In the RT-qPCR method, saliva had 86 positive samples (25.1%) and 256 negative samples (74.8%) and nasopharynx had 93 positive samples (27.1%) and 249 negative samples (72.8%). The agreement between the two tests in saliva and nasopharynx samples was 93% and 94% respectively, based on Cohen’s kappa coefficient (κ) (*P* < 0.001). The rate of sensitivity in this technique was reported at a dilution of 1 × 10^1^ and 100% specificity.

**Conclusions:**

Based on the results of the study the One-Step LAMP assay has multiple advantages. These include simplicity, cost-effectiveness, high sensitivity, and specificity. The One-Step LAMP assay shows promise as a diagnostic tool. It can help manage disease outbreaks, ensure prompt treatment, and safeguard public health by providing rapid, easy-to-use testing.

## Background

 Severe acute respiratory syndrome coronavirus (SARS-CoV) first appeared in China in 2002 [1]. In 2012, a new virus called Middle East Respiratory Syndrome coronavirus (MERS-CoV) emerged in Saudi Arabia. [2]. In late December 2019, an unidentified pneumonia case was reported in Wuhan City, Hubei Province, China [3]. The virus was named SARS-CoV-2 by the World Health Organization (WHO). The International Committee on Taxonomy of Viruses (ICTV) called it SARS-CoV-2 [4, 5]. In terms of morphology, these viruses are similar to the solar corona due to having surface folds, and for this reason, they were named coronavirus [6]. This virus is one of the enveloped, positive-sense, and single-stranded RNA viruses [7, 8]. The human coronavirus causes respiratory diseases. These diseases can range in severity. They include the common cold, pneumonia, and bronchitis. The human coronavirus is called the fastest-changing virus today. It changes quickly because of high-speed nucleotide changes and recombination [9, 10]. This virus contains structural proteins. These proteins include membrane proteins M and E. Additionally, there are nail-shaped proteins called S (spike) [11, 12].

Morphological, serological, and molecular assays are “one of” the methods of diagnosing this virus. The electron microscope is used in the morphological diagnosis method and also, EM be an important tool for diagnosis and research into the ultrastructural basis of disease [13]. However, not all laboratories have this type of microscope. Therefore, it is not a routine diagnostic method. The serological diagnosis method is based on recognizing antibodies and antigens. However, this type of test was used initially during the disease outbreak. Due to changes and mutations in the virus gene, the antigenic structure of the virus constantly changes. As a result, the produced antibody will be different. This leads to low accuracy and sensitivity in these tests, causing false positive or negative results. Today, several rapid point-of-care tests including antigen-based and molecular tests such as Lateral flow assay-based tests, ELISA-based tests, Reverse transcription-quantitative polymerase chain reaction (RT-qPCR), Recombinase Polymerase Amplification (RPA), Reverse transcription loop-mediated isothermal amplification (RT-LAMP), and clustered regularly interspaced short palindromic repeats (CRISPR) used to diagnose SARS-CoV-2 [14–17]. Even though the specificity of these tests is high, the sensitivity of their testing varies from 15 to 95%, depending on various factors such as the status of symptoms, timing of the test, and brands of test. Consequently, point-of-care testing can be used for screening patients suspected of SARS-CoV-2 infection [18]. Therefore, the World Health Organization recommends using molecular methods for diagnosis. Molecular diagnosis methods rely on isolating and multiplying virus nucleic acids. Real-time PCR is a common molecular test for coronavirus diagnosis. It is used in most laboratories [19, 20].

The disease is increasing, so we need a quick and affordable diagnostic method. This method should have high sensitivity and specificity, and focus on rapid molecular detection of the virus. This will help prevent and diagnose the disease quickly. The ELISA test and PCR methods, including RT-PCR and Real-Time PCR, yield results slowly over time. However, the LAMP molecular method is highly accurate and quickly detects the virus. It allows for prompt evaluation and confirmation of the virus in patient samples. This research aims to expedite obtaining accurate results and minimize costs for SARS-CoV-2 tests.

## Materials and methods

### Sample collection

Three hundred forty-two samples were collected from medical centers in Tehran, the Capital of Iran, for this experimental study. The referring patients suspected of SARS-CoV-2 infection were informed of the project’s goals. Written informed consent was obtained from the patients. A questionnaire was filled out. Samples were collected. Nasopharyngeal and saliva samples were taken using a sterile swab. The samples were placed in 2 ml of fresh Viral Transport Media (VTM). Additionally, urine samples were collected from the patients. All samples were transferred to the Cancer Biomedical Research Center while maintaining the cold chain. The Protocols for the collection and transport of specimens were followed. The laboratory received the samples and stored them at -70 °C.

### Ethics approval and consent to participate

The study protocols were approved at the Islamic Azad University, North Tehran branch, and were registered with the ethics code (IR.IAU.TNB.REC.1401.042). The study’s plan was explained to the referring patients first. Written informed consent was obtained from patients who agreed to participate in the study. Patients also completed the questionnaire and then the samples were collected.

### Viral RNA extraction

The sample characteristics were recorded. RNA was extracted from nasopharyngeal, saliva, and urine samples using Biorexfars Co. Iran’s SARS-CoV-2 RNA Extraction Kit. The RNA extraction process had four steps. First, the samples were lysed. Then, the RNA was attached to the filter membrane. Next, the filter was washed and the alcohol was removed. Finally, the RNA was separated from the filter. The samples were initially lysed at 37 °C using Lysis Buffer and Proteinase K. After lysing, the filter attached the RNA, and the washing step eliminated any extra substances, resulting in purified RNA. Finally, the purified RNA was dissolved in an Elution Buffer. Buffers 1 and 2 removed contaminants, but nucleic acids stuck to the filter. Finally, using 100 µl of Elution Buffer, high-purity viral RNA was separated from the filter membrane. To increase RNA concentration, a small volume of Elution Buffer solution was used. The nanodrop machine (NanoDrop 2000c, Thermo Fisher Scientific, USA) measured the purity and quantity of extracted RNA. The purity of RNA was determined based on recommended guidelines. The recommended guidelines consider a ratio of 260/280 ~ 2.0 as adequate and acceptable. The extracted RNA samples were stored at -70 °C for subsequent steps. All the steps mentioned in RNA extraction took around 30 min.

### One‑step RT‑qPCR

The assay aimed to detect SARS-CoV-2. It was part of routine testing for suspected patients. To conduct the One-Step RT-qPCR assay using the COVID-19 kit (Pishtaz Teb Co., Iran/ PT. COVID.100) a reaction volume of 20 µl was prepared. This consisted of 5 µl of RNA template, 9 µl of resuspended master mix, 2 µl of the N/ICON Primer & probe mix (HEX/ROX), and 5 µl of RNase-free water. The tubes were centrifuged to eliminate air bubbles and then moved to the amplification area. The Rotor-Gene Q-Pure Detection Real-Time PCR system was used in this research (Rotor-Gene Q MDx – QIAGEN, USA). The HEX channel (N gene) and ROX channel (RNase P) were used as the internal control to detect SARS-CoV-2 RNA. Cycles were conducted for 20 min at 50 °C to reverse transcribe. The process started with an initial cDNA denaturation at 95 °C for 3 min followed by 45 cycles of denaturation at 95 °C for 15s, annealing, and extension with fluorescence measurement at 55 °C for 40s. The final step involved cooling at 25 °C for 10.

### LAMP primer design

LAMP primers were designed for the N gene (NC_045512.2) (28274.29533) of the SARS-CoV-2 virus (Table 1). Primer Explorer V5 software (https://primerexplorer.jp/e/*)* was used to design a set of 6 primers includes a pair of external primers (F3 = 18nt and B3 = 20nt), a pair of internal two-part primers (FIP: F1C = 18nt, F2 = 19nt) and (BIP: B1C = 21nt, and B2 = 20nt) and a pair of loop primers (LF = 21nt and LB = 20nt) that identify 8 regions on the target sequence. The Primer BLAST method in NCBI checked the specificity of SARS-CoV-2 primers. It confirmed that the primers were completely specific, and no other targets were found in the selected database. Also, to check alignment and specific regions for LAMP primers in SARS-CoV-2 CLC Genomics Workbench 8.5 software was used. The target sequences for alignment include five SARS-CoV-2 sequences including MN938384, MN988713, MN985325, MN908947 and MN975262 and seven SARS-CoV sequences NC_004718, AY613947, AY313906, AY559094, AY502924, AY278491 and AY502927.

**Table 1:** Characteristics of primers used for the one-step RT-LAMP technique

Name of Primer set *N* (28274.29533)	Sequence (5´–3´)	Length of primer(nt)
**F3**	**GCCATAGGCTTCTACGTC**	**18**
B3	**TTGCTCTCAGGCTGGATCAG**	**20**
**FIP**	**TGCGTACTGCTGCCTGGA – CGCTGACACGCCTCATCTG**	**F1C = 18 F2 = 19**
**BIP**	**TCTCCAGGTAGTATGCTTGGC - ATGTGTCAAGCAGCAGTATG**	**B1C = 21 B2 = 20**
**LF**	**TGTTCCGACTACCAGATGAGC**	**21**
**LB**	**ATGATGGTGATGCAGCTGTG**	**20**

### One‑step RT-LAMP

**Fig. 1:**
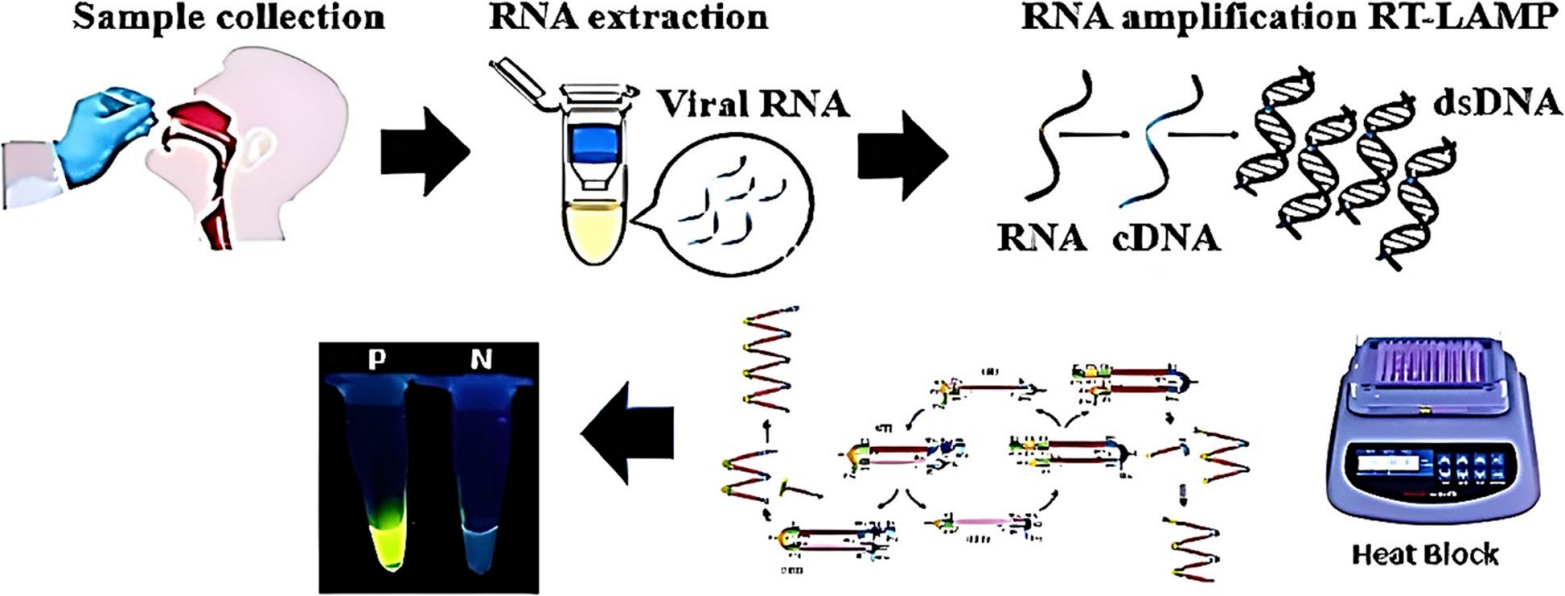
Stages of Identification of SARS-CoV-2 utilizing the One-Step LAMP method. Positive control (P) Negative control (N)

The One-Step LAMP assay was conducted in a 25 µl reaction mixture, containing 5 pmol of each F3 and B3 external primers, 40 pmol of each FIP and BIP internal primers, 20 pmol of each LF and LB loop primers, and 8U (1 µl) of *Bst DNA/RNA Polymerase* 3.0 (8 U/ µl, New England Biolabs, USA), the revised version of the key enzyme utilized in the LAMP reaction to amplify RNA directly in a buffer of 2.5 µl [20 mM Tris–HCl (PH 8.8), 8 mM MgSO4, 10 mM (NH4)2SO4, 0.1% Tween 20], 10 mM KCl, 0.8 M betaine (Sigma-Aldrich), 1.4 mM deoxynucleoside triphosphates (dNTP), and 1 µl of template RNA. The genomic RNA of the standard SARS-CoV-2 strain (Delta-b.1.617.2) and double-deionized distilled water were used as the positive and negative controls in each run respectively. The reaction mixture was incubated at 65 °C for 30 min in a water bath and then was deactivated by placing it in an 80 °C incubator for 2 min. Finally, to the visual detection of the amplicons, 3 µl of EVA Green (Invitrogen Carlsbad, CA, USA) diluted to a 1:10 ratio from a 10,000 × concentration was added to the reaction tubes. In the presence of LAMP amplification products, the solution turned green while, in the absence of the amplicon, it stayed colorless. To confirm the results, 10 µl of the LAMP products were electrophoresis on a 1.5% agarose gel (Invitrogen Co, USA) stained with DNA-safe stain (Cinagen Co, Iran) (Fig. 1).

### Clinical and analytical sensitivity and specificity of the one‑step RT-LAMP assay

The One-Step LAMP assays analytical sensitivity was evaluated using SARS-CoV-2 RNA. The RNA standard underwent tenfold serial dilutions from 1 × 10^7^ to 1 × 10^−2^ in 1X HBSS (Merk, 55,021 C). The analytical sensitivity results were confirmed by repeating the tests three times. Additionally, to ensure accuracy, the reaction product was evaluated by electrophoresis and assessed under UV light using gel documentation. This method avoids visual errors and verifies the analytical sensitivity test results. The clinical sensitivity of the One-Step LAMP assays was determined using the optimized protocol. A total of 20 positive and 20 negative clinical samples were selected. These samples had previously been tested by RT-qPCR.

Various genetically related viruses, including Influenza A virus, Influenza B virus, Respiratory syncytial virus, Adenovirus, *E. coli*, and *Toxoplasma gondii*, were used to examine the analytical specificity of the One-Step LAMP assay. Moreover, to check for the asymptomatic carriers, 55 suspicious samples (36 salivae and 19 nasopharynx) (ct between 30 and 35) from individuals without common symptoms of the disease were evaluated.

### Relationship between variables and SARS-CoV-2 infection

The One-Step RT-qPCR assay was utilized to examine how certain variables are related to the presence of SARS-CoV-2 infection. This test is widely recognized as the most reliable method for diagnosing SARS-CoV-2 virus in individuals who are suspected of being infected.

### Statistical analysis

The data analysis was conducted using SPSS ver. 22 for Windows, which used frequency distribution tables to describe the data. Statistical tests, like Chi-square (χ2) and Fisher exact tests, determined the probable significant statistical relationship between variables. Cohen’s kappa coefficient (κ) calculated the agreement of the molecular tests. The clinical and analytical sensitivity and specificity of the one‑step RT-LAMP assay were measured by using the optimized protocol applied on a total of 40 positive and negative clinical samples and SARS-CoV-2 RNA standard tenfold serial dilutions from 1 × 10^7^ to 1 × 10^−2^ in 1X HBSS as well as the templates of various genetically related viruses in triplicated. Multivariate modeling of the data was carried out using logistic regression, and after adjustments, associations were tested using odd ratios (OR) and 95% confidence intervals (CI). In cases where the expected frequencies were less than five, the statistical significance was calculated using the Monte Carlo method simulation based on 10,000 replicates. The statistically significant level in all tests was considered to be 0.05.

## Results

### One‑step RT‑qPCR and one‑step RT-LAMP

In this study, the one-step RT-LAMP method detected SARS-CoV-2 in 3 samples: positive saliva 92(26.9%), negative saliva 250(73.09%), positive nasopharynx 94(27.4%), negative nasopharynx 248(72.51%) and positive urine 1(0.2%), negative urine 341(99.7%). The one-step Real-Time PCR method detected SARS-CoV-2 in 2 samples: positive saliva 86(25.1%), negative saliva 256(74.8%) and positive nasopharynx 93(27.1%), negative nasopharynx 249(72.8%). None of the urine samples were positive for SARS-CoV-2 using one-step Real-Time PCR (Table 2).

**Table 2:** Positive samples in RT-LAMP and real-Time-PCR (NA: Nasopharynx / SA: saliva / U: urine)

Samples	NA	SA	U
Methods			
**RT-LAMP**	94(27.4%)	92(26.9%)	1(0.2%)
**RT-qPCR**	93(27.1%)	86(25.1%)	0(0%)

Based on Cohen’s kappa coefficient (κ), the agreement between the two tests was 93% in the saliva sample and 94% in the Nasopharynx samples. These percentages were statistically significant (*P* < 0.001) (Table 3). No statistics were computed for urine samples. Both RT-LAMP and Real-Time PCR were constant, and the results were negative.

**Table 3:** The consensus of the one-step RT-qPCR test and one-step LAMP technique results in the diagnosis of SARS-CoV-2

			**One-Step RT-qPCR saliva**			**Total**	
			**Negative**	**Positive**			
**One-Step RT-LAMP Saliva**	**Negative**	Count	250	0	250		*P* < 0.001 Kappa = 93%
		% of Total	73.0%	0%	73.0%		
	**Positive**	Count	6	86	92		
		% of Total	1.7%	25.1%	26.8%		
**Total**		Count	256	86	342		
		% of Total	74.8%	25.1%	100.0%		
			**One-Step RT-qPCR-** **Nasopharyngeal**		**Total**		
			**Negative**	**Positive**			
**One-Step LAMP Nasopharyngeal**	**Negative**	Count	248	0	248		*P* < 0.001 Kappa = 94%
		% of Total	72.5%	0%	72.5%		
	**Positive**	Count	1	93	94		
		% of Total	0.2%	27.19%	27.4%		
**Total**		Count	249	93	342		
		% of Total	72.71%	27.19%	100.0%		

**Fig. 2:**
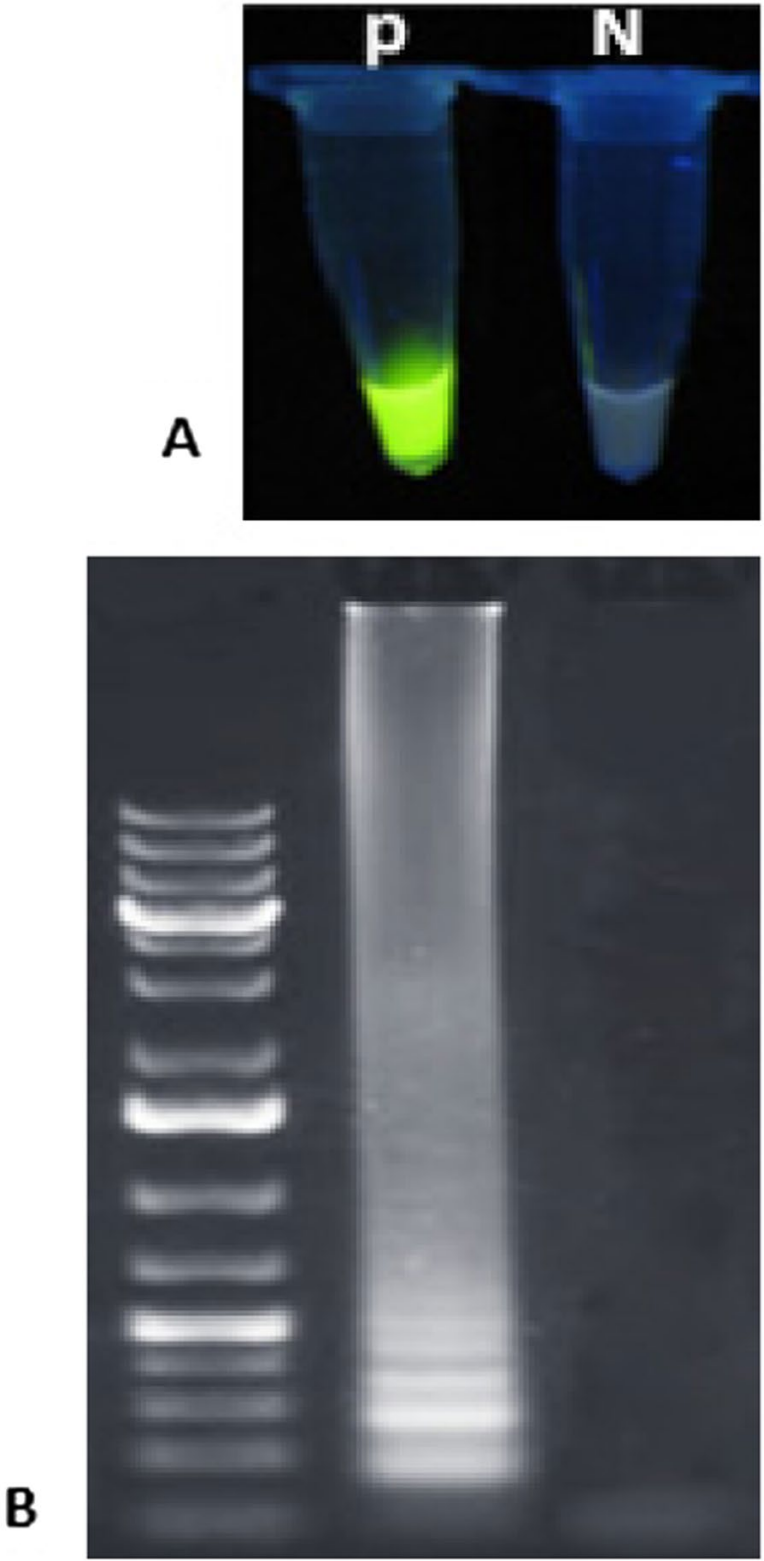
Monitoring of LAMP amplification of the SARS-CoV-2 RNA. **A** Visual inspection of the RNA amplification by fluorescence of the reaction mixture under normal light. **B** Agarose gel analysis of LAMP amplified product. Positive control (P); Negative control (N) (100 bp molecular weight marker)

The primers and probes in the RT-qPCR kit were designed based on the conserved sequence of the novel coronavirus (SARS-CoV-2) per the manufacturer’s protocol. They have a high detection rate of the target gene fragment. Also, the RT-qPCR kit had no cross-reactions among positive samples of coronavirus (NL63, HKU1, 229E, OC43). In the One-Step LAMP assay, the new version of the key enzyme of the LAMP reaction, *Bst DNA Polymerase* 3, was used successfully (Fig. 2A and B).

### Clinical and analytical sensitivity and specificity of the one-step LAMP assays

**Fig. 3:**
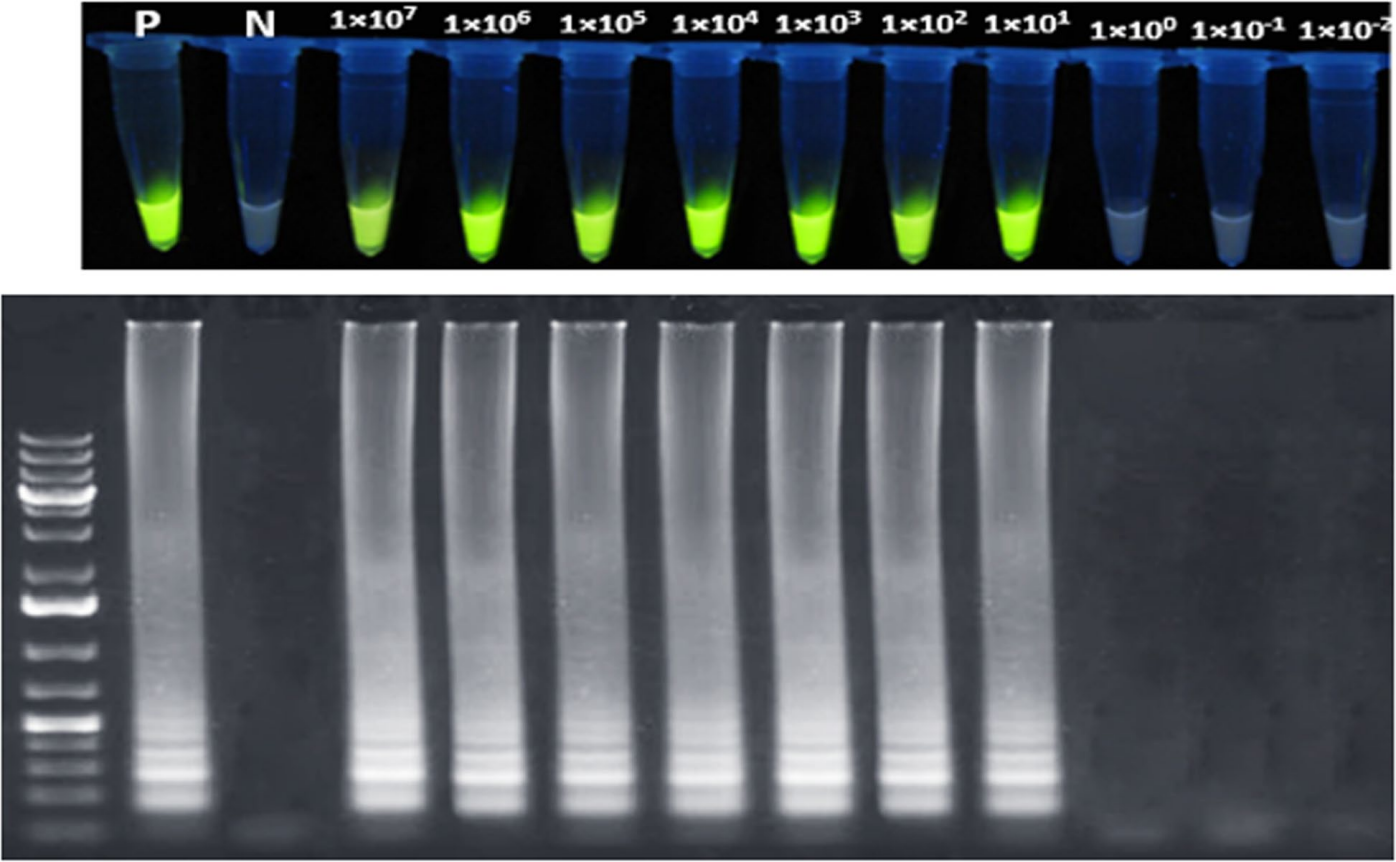
Analytical sensitivity of the One-step LAMP assay for the detection of SARS-CoV-2 RNA based on the N gene amplification. Tenfold serial dilutions of the RNA standard of SARS-CoV-2 from 1 × 10^**7**^ to 1 × 10^−2^ copies per reaction

A series of dilutions of the RNA standard ranging from a concentration of 1 × 10^7^ to 1 × 10^−2^were made to check how well One-Step LAMP assays can detect SARS-Cov-2 RNA. The assay’s analytical sensitivity was determined to be able to detect a 1 × 10^1^ dilution of the RNA standard per reaction in all three trials. The accuracy of the results was confirmed by examining the products of the LAMP reaction through electrophoresis on a 1.5% agarose gel and using a gel documentation system (Fig. 3). The optimized One-Step LAMP protocol showed consistent results across all positive and negative clinical samples from patients, indicating that it was reliable. The positive controls consistently produced positive results, while non-SARS-CoV-2 templates produced negative results, demonstrating that the assay had 100% specificity. Additionally, six special primers were used in the LAMP test. This made it completely specific which only detects the SARS-CoV-2 genome. No specificity test was performed for SARS-CoV and Mers-Coronavirus due to the limited access.

Regarding asymptomatic carriers, all 55 samples were reported negative in the initial test with both RT-LAMP and RT-qPCR methods. After 48 h, all 55 cases were negative in the repeat RT-qPCR test. However, in the repeat RT-LAMP test, 6 out of 55 people tested positive and still had no symptoms. After 1 week of following these 6 LAMP-positive cases, clinical symptoms were seen in these people.

### Possible relations between variables and SARS-CoV-2 infection

The One-Step RT-qPCR assay was used to evaluate the relationship between certain variables and the presence of SARS-CoV-2 infection. This assay is the most common and accurate test for diagnosing SARS-CoV-2 infection among individuals suspected of being infected. In total, 86 positive samples were reported using the RT-qPCR technique. In terms of age, the mean age of participants with One-Step RT-qPCR test results was 43.24 ± 17.21. There was no statistically significant relationship between age and SARS-CoV-2 infection (*P* > 0.05). Also, regarding gender, out of 86 people with the virus, *P* < 0.001 shows a statistically significant difference between gender and virus infection. Statistical analysis of data using the Chi-square test showed that there was a statistically significant relationship between SARS-CoV-2 infection using the one-step RT-qPCR test and clinical symptoms of fever (*P* < 0.001), headache (*P* < 0.001), and cough (*P* = 0.001). While the clinical signs of diarrhea and vomiting (*P* = 0.821), Body aches (*P* = 0.92), Sore throat (*P* = 0.65), and loss of taste and smell (*P* = 0.63), Shortness of breath (*P* = 0.42) had a remarkable but insignificant relationship. There was a significant relationship between SARS-CoV-2 infection and diabetes using the chi-square test (*P* < 0.001) (Table 4).

**Table 4:** SARS-CoV-2 infection and demographic variables

**Variables**		One-Step RT-LAMP				*P* value	Odds Ratio (CI %95)	One-Step RT-qPCR				*P* value	Odds Ratio (CI %95)
		Negative *N* (%)		Positive *N* (%)				Negative *N* (%)		Positive *N* (%)			
		NA	SA	NA	SA			NA	SA	NA	SA		
Gender	**Male**	**195 (72.7)**	**212(79.1)**	**73 (27.2)**	**56 (20.8)**	**0.001**	**1.2** **(0.863–1.917)**	**197(73.5)**	**214(79.8)**	**71 (26.4)**	**54(20.1)**	0.001	**1.34** **(0.35–1.79)**
	**Female**	**53 (71.6)**	**38 (51.3)**	**21 (28.3)**	**36 (48.6)**			**52(70.2)**	**42(56.7)**	**22 (29.7)**	**32(43.2)**		
Age category	**≤ 18**	**22 (62.8)**	**22 (62.8)**	**13 (37.1)**	**13 (37.1)**	**0.05**	**6.89** **(0.84–56.82)**	**22 (62.8)**	**23 (65.87**	**13 (37.1)**	**12 (34.2)**	**0.583**	**7.35** **(0.89–60.63)**
	**19–64**	**191 (74.6)**	**193(75.3)**	**65 (25.3)**	**63(24.6)**			**191(74.6)**	**197(76.9)**	**65 (25.3)**	**59(23.1)**		
	**≥ 65**	**35 (68.6)**	**35 (68.6)**	**16 (31.3)**	**16 (31.3)**			**36 (70.5)**	**36 (70.5)**	**15 (29.4)**	**15 (29.4)**		
Clinical symptoms	**Fever**	**14 (30.4)**	**16 (34.7)**	**32 (69.5)**	**30 (65.2)**	**0.001**	**3.72** **(1.45–9.56)**	**14 (30.4)**	**19 (41.3)**	**32 (69.5)**	**27 (58.6)**	0.001	**2.10** **(0.63–2.42**)
	**Body aches**	**13 (81.2)**	**13 (81.2)**	**3 (18.7)**	**3 (18.7)**	**0.923**	**3.84** **(0.42–34.86)**	**13 (81.2)**	**14 (87.5)**	**3 (18.7)**	**2 (12.5)**	**0.228**	**4.28** **(0.71–5.21)**
	**Headache**	**4 (18.1)**	**4 (18.1)**	**18 (81.8)**	**18 (81.8)**	**0.001**	**1.08** **(0.62–3.19)**	**5 (22.7)**	**4 (18.1)**	**17 (77.2)**	**18 (81.8)**	0.001	**3.29** **(0.74–8.54)**
	**Sore throat**	**15 (78.9)**	**15 (78.9)**	**4** **(21)**	**4** **(21)**	**0.659**	**2.3** **(1.12–5.04)**	**15 (78.9**	**15 (78.9)**	**4** **(21))**	**4** **(21)**	**0.932**	**2.62** **(1.57–4.16)**
	**Cough**	**8 (27.5)**	**8 (27.5)**	**21 (72.4)**	**21 (72.4)**	**0.001**	**1.97** **(0.27–5.46)**	**8 (27.5)**	**8 (27.5)**	**21 (72.4)**	**21 (72.4)**	0.001	**1.74** **(1.13–2.97)**
	**Loss of smell & and taste**	**19 (90.4)**	**19 (90.4)**	**2 (9.5)**	**2 (9.5)**	**0.633**	**3.46** **(1.95–6.28)**	**19 (90.4)**	**19 (90.4)**	**2 (9.5)**	**2 (9.5))**	**0.857**	**1.31** **(1.22–5.21)**
	**Diarrhea & Vomiting**	**5 (83.3)**	**5 (83.3)**	**1 (16.6)**	**1 (16.6)**	**0.821**	**1.67** **(0.89–3.1)**	**5 (83.3)**	**5 (83.3)**	**1 (16.6)**	**1 (16.6)**	**0.365**	**5.37** **(1.93–7.28)**
	**Shortness of breath**	**9 (60)**	**9 (60)**	**6** **(22)**	**6** **(22)**	**0.425**	**1.21** **(0.7–2.11)**	**9 (60**	**9 (60)**	**6** **(22)**	**6** **(22)**	**0.964**	**4.39** **(1.78–6.47)**
	**Asymptomatic**	**161 (95.8)**	**161(95.8)**	**7 (4.1)**	**7 (4.1)**	**0.797**	**1.51** **(0.87–2.62)**	**161(95.8)**	**163(97)**	**7 (4.1)**	**5 (2.9)**	**0.458**	**2.94** **(1.02–4.78)**
Underlying disease	**Diabetic patients**	**5 (12.8)**	**7 (17.9)**	**34 (81.1)**	**32 (82.1)**	**0.001**	**3.5** **( 0.78–9.51)**	**5 (12.8)**	**8 (20.5)**	**34 (81.1)**	**31 (79.4)**	0.001	**1.41** **(1.09–7.21)**
	**Kidney diseases**	**10 (83.3)**	**10 (83.3**	**2 (16.6)**	**2 (16.6)**	**0.358**	**2.16** **(1.56–3.37)**	**10 (83.3)**	**10 (83.3)**	**2 (16.6)**	**2 (16.6)**	**0.712**	**3.19** **(1.08–4.16)**
	**Pulmonary diseases**	**9 (64.2)**	**9 (64.2)**	**5 (35.7)**	**5 (35.7)**	**0.145**	**1.5** **(0.11–5.45)**	**9 (64.2)**	**9 (64.2)**	**5 (35.7)**	**5 (35.7)**	**0.145**	**1.31** **(1.00-2.37)**
	**Cardiovascular diseases**	**12 (75)**	**12 (75)**	**4** **(23)**	**4** **(23)**	**0.564**	**1.06** **(0.35–3.17)**	**13 (81.2)**	**12 (75)**	**3 (18.7)**	**4** **(23)**	**0.695**	**1.47** **(1.07–7.12)**
	**Cancer disease**	**2 (66.6)**	**2 (66.6)**	**1 (33.3)**	**1 (33.3)**	**0.963**	**1.03** **(0.43–2.42)**	**2 (66.6)**	**2 (66.6)**	**1 (33.3)**	**1 (33.3)**	**0.554**	**3.96** **(0.43–5.63)**
	**No underlying conditions**	**210 (81.3)**	**210 (81.3**	**48 (18.6)**	**48 (18.6)**	**0.714**	**1.99** **(1.07–3.72)**	**210(81.3)**	**215(82.1)**	**48 (18.6)**	**43 (16.6)**	**0.974**	**2.01** **(0.25–47.1)**

## Discussion

In early January 2020, a new coronavirus was identified as the infectious agent causing an outbreak of viral pneumonia, the first case was reported in Wuhan, China in December 2019 [21, 24]. According to the prediction of the World Health Organization, the prevalence of this disease is still increasing and by 2030 it will be known as the third cause of death in the world [9, 25]. This disease has a unique complexity and has multiple dimensions and consequences that can contribute to significant health, social, and economic costs for individuals, communities, and health services [26]. Also, the rapid and timely diagnosis of the coronavirus is essential due to the high rate of spread of the virus, so there is a need for a fast and cheap diagnostic method that can be performed in different places and is affordable with high sensitivity and specificity [23]. The LAMP technique is one of the molecular diagnosis methods that was optimized for the first time in 2000 by Notomi and her colleagues, and since then there have been studies related to the applications of this technique [27]. This technique has been used to detect many pathogens such as herpes simplex virus, influenza and other pathogens in various studies [28, 29]. The Real-time PCR technique is one of the most common molecular detection methods for SARS-CoV-2. Although it is relatively sensitive and accurate, many diagnostic centers lack the advanced equipment required to use it. As a result, its use is limited to specialized centers. Therefore, the LAMP technique, a fast and reliable molecular diagnostic method, is gaining popularity. Unlike the Real-time PCR technique, it does not require expensive equipment and this technique can be used in areas with limited facilities [30] (Table 5). In the current study of the one-step LAMP technique, we were able to optimize the LAMP technique for the detection of the SARS-CoV-2 virus by using specific primers designed for the N region, and in terms of sensitivity and specificity was compared with the Taq Man, One-Step RT-qPCR. The nucleocapsid (N) gene region is often included in molecular diagnostic tests for viral infections, including SARS-CoV-2 because it is a highly conserved region of the viral genome and is abundant in mRNA. Therefore, detecting the N gene region can help to confirm the presence of the virus in a patient sample and aid in the diagnosis of the infection [31]. Based on Cohen’s kappa test, the agreement rate between LAMP and RT-qPCR tests in the nasopharyngeal sample was 94% (27.4% and 27.1% positivity rate respectively) and 93% in the saliva sample (26.8% and 25.1% positivity rate respectively) which, was statistically significant (*P* < 0.001). The results were consistent with the results in the study by Khanizadeh et al. [32] in terms of the agreement between the two techniques showing the accuracy and reliability of the results of the One-Step LAMP technique compared to the One-Step RT-qPCR method. Several studies have reported that the LAMP technique is a simple, fast, and sensitive isothermal amplification technique. It is characterized by its reduced dependence on sophisticated equipment. Moreover, the LAMP method has demonstrated great success in identifying and diagnosing SARS-CoV-2 infection in clinical samples from patients because of highly specific on six independent primers to recognize the target sequence [33, 34]. In this study, it was shown that the One-Step LAMP technique was able to detect 1×10^1^ copies of the standard RNA of the SARS-CoV-2 virus, which shows the high sensitivity of this technique so the results were consistent with the results in the studies of Khanizadeh et al [32]. The high sensitivity of this technique can be attributed to the use of two forward and reverse loop primers (LF and LB primers), which act as an accelerator of the replication reaction, reducing the reaction time and increasing the detection speed. Also, due to the use of 4–6 primers, which can detect 6–8 regions of the target sequence, the specificity of this technique is 100% [33–35]. One of the advantages of the One-Step LAMP technique compared to Real-time PCR is its simplicity and one-step process, which is capable of amplifying a large number of RNA copies (10^9^) in less than an hour under isothermal conditions [36, 37].

**Table 5:** Advantages and disadvantages of real-Time-PCR and RT-LAMP techniques

	Real-Time- PCR	RT-LAMP
Advantage	• Simultaneous amplification and detection during exponential amplification • Real-time monitoring of amplification as it happens • Quantitative, thus useful for monitoring the viral load • Lower carry-over contamination due to closed tube operation • Increased sensitivity due to fluorescent chemistry • High throughput analysis due to software driven operation	• Isothermal field-based gene amplification without requiring thermal cycle Amplification can be accomplished with water bath/heating block Real-time as well as quantitative • Higher amplification efficiency and sensitivity • Naked eye visual monitoring either through turbidity or color change by fluorescent intercalating dye
Disadvantage	• Expensive detection equipment and consumable • Requirement for fluorescent probe • Restricted to referral laboratory with good financial support	• Complicated primer design (a requirement for six primers) • Two long primers of HPLC grade purity • Restricted availability of reagents and Equipment in some countries • Laboratory based

The routine samples used in most medical centers for the diagnosis of SARS-CoV-2 are nasopharyngeal specimens. However, sampling from the nasopharynx can be invasive and may cause discomfort or non-cooperation from patients. Additionally, patients may sneeze due to the stimulation of the nasal passages, leading to the spread of the virus in the environment and the contamination of the sampling personnel and those around them. On the other hand, saliva and urine do not require a certified swab, specific collection receptacle, or transport media, and do not have to be obtained by a skilled healthcare provider [38, 39]. For these reasons, in addition to examining nasopharyngeal samples, saliva, and urine samples were also used in this study. In the present study, the sensitivity of SARS-CoV-2 detection in saliva and nasopharynx samples was reported as 88.2% and 92.9%, respectively, the results were consistent with the results of Sazed et al [40]. Based on the obtained results in the current study, the RT-LAMP method can identify the virus carriers, if the RT-qPCR method proves otherwise. However, RT-qPCR may fail to detect the virus and produce false-negative results, particularly if the viral load is very low in the patient’s sample. This can occur in patients who have recently been infected, or in patients who are in the early stages of the disease [22, 40]. As in the current study, in the investigation of 55 asymptomatic people suspected of SARS-CoV-2 infection, all 55 cases were negative in the repeat RT-qPCR test after 48 h. However, in the repeat RT-LAMP test, 6 out of 55 people tested positive and still had no symptoms. After 1 week of following these 6 LAMP-positive cases, clinical symptoms were seen in these people. Then, due to the high virus load, the positivity of the 6 cases (ct < 30) was confirmed with the gold standard RT-qPCR method, while in low virus load, the RT-qPCR method could not detect the virus. According to the obtained results, it seems that the RT-LAMP method was able to detect SARS-CoV-2 carriers, one week before emerging the clinical symptoms, while the RT-qPCR method is not able to detect carriers. In this study, there was no significant relationship between age and SARS-CoV-2 infection that was consistent with the results of the studies by Khanizadeh et al [32], and Novosad et al [41]. While in contrary to this a significant relationship between age and SARS-CoV-2 infection was reported in the study of XU et al [42]. In terms of the gender, a significant difference was observed in the present study (*P* < 0.001), so the prevalence of infection was reported to be higher in men than in women, and the reason for this can be attributed to different genetic, physiological, and hormonal causes in men and women. This result was consistent with the results reported in the study by Paschou et al [43]. Also, Based on various research in terms of the significant relationship between the underlying diseases and the severity of the SARS-CoV-2 disease, it has been shown that the highest rate of infection is related to diabetic patients, which is one of the important reasons for the severity of the SARS-CoV-2 in diabetic patients due to the increased expression of ACE2 as a receptor for spike virus, in all tissues, especially lungs [44, 45]. In the present study, in terms of people with underlying diseases, diabetic patients included the highest amount of other underlying diseases, and the results of our study were consistent with the results of the study by Qiao Shi et al [46]. In this research, based on the univariate logistic regression test, diabetic patients had approximately 3 times higher odds of being infected with SARS-CoV-2 compared to individuals without diabetes. In terms of clinical symptoms, factors such as age, genetics, gender, and immune system in different people can play a very important role in the severity of clinical symptoms. In this study, one of the most common clinical symptoms in many people with SARS-CoV-2 was cough and fever. Statistical results showed that there is a significant relationship between SARS-CoV-2 infection and the symptoms of fever, cough, and headache, while it was not significantly associated with symptoms of diarrhea and vomiting, sore throat, and body aches. In a similar study, Sarker et al [47] reported that the most common symptoms in patients with SARS-CoV-2 included fever and cough. The study found that the One-Step LAMP assay is an effective technique for detecting SARS-CoV-2 in suspected individuals, with advantages over the One-Step RT-qPCR assay in terms of simplicity, cost, sensitivity, and specificity. These findings suggest that the One-Step LAMP assay has great potential as a valuable diagnostic tool for controlling disease epidemics, providing timely treatment, and protecting public health.

**Fig. 4:**
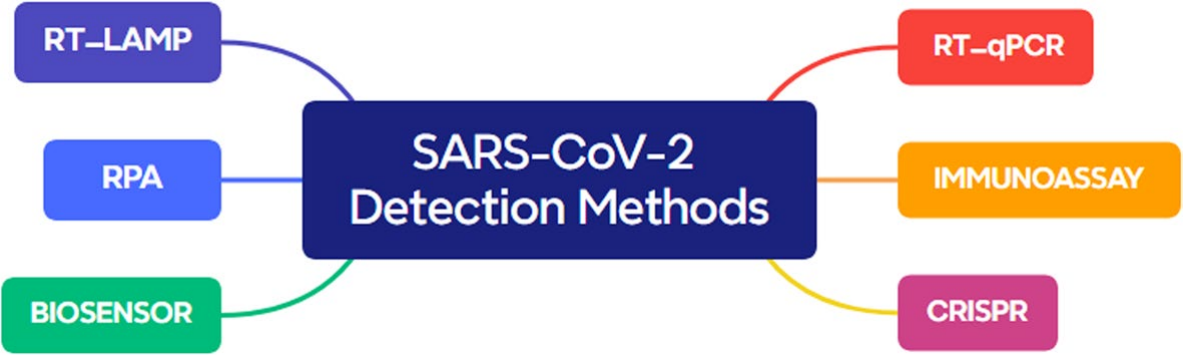
Diagnostic methods of SARS-CoV-2

In addition to RT-LAMP and RT-PCR tests, there are different point-of-care tests, such as RPA (Recombinase Polymerase Amplification), biosensors, immunoassays, and the CRISPR/Cas system, for the diagnosis of SARS-CoV-2 (Fig. 4).

RPA (Recombinase Polymerase Amplification) is an isothermal amplification technique that uses recombinase enzymes and specific primers to amplify the target DNA or RNA. It operates at a constant temperature and can rapidly amplify the target sequence. RPA assays can provide results within 15–30 min, making them suitable for point-of-care testing also, RPA can be performed using portable, battery-operated devices, allowing testing in decentralized settings. About sensitivity and specificity, RPA has demonstrated high sensitivity and specificity for the detection of SARS-CoV-2. In terms of limitations, RPA typically requires a dedicated device to maintain the constant temperature, which may limit accessibility in certain settings, and also, the cost of RPA assays can vary depending on the specific protocols and availability of equipment [48].

Biosensors are another point-of-care test. Biosensors are devices that detect specific biological targets by measuring the interaction between the target and a bioreceptor, such as antibodies or nucleic acids. For SARS-CoV-2, biosensors can detect viral proteins or genetic material. Biosensors can provide results within minutes to hours, making them suitable for point-of-care testing. Some biosensors are designed as handheld devices, enabling on-site testing. Biosensors Sensitivity and specificity can achieve high sensitivity and specificity, depending on the design and bioreceptor used. In terms of limitations, biosensor development can be complex, requiring optimization of the bioreceptor and signal transduction mechanisms also biosensors need specific bioreceptors for the target of interest, which may limit their utility if the target undergoes significant mutations [49].

Immunoassay techniques detect the presence of specific antibodies or antigens in a sample. They can utilize various formats, such as lateral flow assays or enzyme-linked immunosorbent assays (ELISA). Some immunoassays can provide results within minutes to hours, allowing for point-of-care testing. Immunoassays can be relatively simple to perform and interpret, and some formats do not require specialized equipment also immunoassays are well-established and widely used in diagnostics. The performance of immunoassays can vary, and some formats may have limitations in terms of sensitivity and specificity compared to molecular tests, and also immunoassays rely on the presence of antibodies, which may take time to develop after infection. Early-stage infections may yield false-negative results [50].

The CRISPR/Cas system has been adapted for diagnostic purposes, where it can be used for the detection of specific nucleic acid sequences, including SARS-CoV-2 RNA. CRISPR-based assays can provide results within minutes to hours, allowing for point-of-care testing. CRISPR-based assays can offer high specificity for the target sequences but may require specialized equipment for detection, such as fluorescence readers or lateral flow strips also the implementation of CRISPR-based assays can be technically challenging and may require trained personnel. CRISPR-based diagnostic assays are still in the early stages of development and may require further optimization and validation [51].

It’s important to note that the choice of RNA extraction method can impact the downstream diagnostic process, including the sensitivity and accuracy of the test. The selection of the most appropriate RNA extraction technique depends on factors such as sample type, throughput, available resources, and the specific diagnostic platform being used. When comparing the time required for RNA extraction among different techniques, including RT-LAMP, RT-PCR, RPA, and CRISPR/Cas, it’s important to consider that the extraction time can vary depending on the specific method used and the complexity of the workflow. The time required for RNA extraction in different methods can vary depending on the chosen extraction method, ranging around from 20 to 60 min in RT-LAMP, RPA, CRISPR/Cas and RT-PCR [52].

In summary, each of these point-of-care tests has its advantages and limitations. RPA offers rapid results and portability but requires dedicated equipment. Biosensors provide rapid results and portability, but development can be complex. Immunoassays are simple and cost-effective but may have limitations in sensitivity and specificity. CRISPR-based assays offer high specificity but may require specialized equipment and further development. The choice of test depends on factors such as time, resource availability, technical expertise, and specific testing requirements.

The lack of access to different real-time PCR kits with different brands for comparison to the RT-LAMP technique was one of the limitations of our study.

## Conclusions

To summarize the findings, the study concluded that the One-Step LAMP assay is an accurate, rapid, and effective method for detecting SARS-CoV-2 in individuals suspected of infection, particularly in resource-limited or underdeveloped countries. This technique offers advantages over the TaqMan One-Step RT-qPCR, including its simplicity, affordability, sensitivity, and specificity. As a result, the One-Step LAMP assay holds significant potential as a valuable diagnostic tool for controlling disease outbreaks, ensuring timely treatment, and safeguarding public health, particularly in low-resource settings.

## Data Availability

All data generated or analyzed during this study are included in this published article.

## Abbreviations

LAMP: Loop-mediated isothermal amplification
SARS-CoV-2: Severe Acute Respiratory Syndrome Coronavirus 2
FIP: Forward internal primer
BIP: Backward internal primer
VTM: Viral transport media
dNTP: deoxynucleoside triphosphates
